# Supplementary material for: Bothrops lanceolatus snake venom impairs mitochondrial respiration and induces DNA release in human heart preparation
Source: PLoS Negl Trop Dis. 2022 Jun 21;16(6):e0010523. doi: 10.1371/journal.pntd.0010523 (PMC9249236; doi:10.1371/journal.pntd.0010523)
Supplement: Attachment — Submitted filename: Reviewers PLOS Neglected Tropical Diseases.docx [file pntd.0010523.s003.docx]

**View Letter**

**Decision on your PLOS Neglected Tropical Diseases submission (PNTD-D-22-00263) - [EMID:0f0e188223a792c7]**

**sam. 16/04/2022 08:15**

**60-day due date**

Dear Pr NEVIERE,
 
Thank you very much for submitting your manuscript "Bothrops lanceolatus snake venom impairs mitochondrial respiration and induces DNA release in human heart preparation" for consideration at PLOS Neglected Tropical Diseases. As with all papers reviewed by the journal, your manuscript was reviewed by members of the editorial board and by several independent reviewers. In light of the reviews (below this email), we would like to invite the resubmission of a significantly-revised version that takes into account the reviewers' comments.
  
We cannot make any decision about publication until we have seen the revised manuscript and your response to the reviewers' comments. Your revised manuscript is also likely to be sent to reviewers for further evaluation.
 
When you are ready to resubmit, please upload the following:

[1] A letter containing a detailed list of your responses to the review comments and a description of the changes you have made in the manuscript. Please note while forming your response, if your article is accepted, you may have the opportunity to make the peer review history publicly available. The record will include editor decision letters (with reviews) and your responses to reviewer comments. If eligible, we will contact you to opt in or out.

[2] Two versions of the revised manuscript: one with either highlights or tracked changes denoting where the text has been changed; the other a clean version (uploaded as the manuscript file).

Important additional instructions are given below your reviewer comments.

Please prepare and submit your revised manuscript within 60 days. If you anticipate any delay, please let us know the expected resubmission date by replying to this email. Please note that revised manuscripts received after the 60-day due date may require evaluation and peer review similar to newly submitted manuscripts.
 
Thank you again for your submission. We hope that our editorial process has been constructive so far, and we welcome your feedback at any time. Please don't hesitate to contact us if you have any questions or comments.

Kartik Sunagar, Ph.D.
Deputy Editor
PLOS Neglected Tropical Diseases

**Reviewer's Responses to Questions**

**Key Review Criteria Required for Acceptance?**

As you describe the new analyses required for acceptance, please consider the following:

**Methods**

- Are the objectives of the study clearly articulated with a clear testable hypothesis stated?
- Is the study design appropriate to address the stated objectives?
- Is the population clearly described and appropriate for the hypothesis being tested?
- Is the sample size sufficient to ensure adequate power to address the hypothesis being tested?
- Were correct statistical analysis used to support conclusions?
- Are there concerns about ethical or regulatory requirements being met?

**Reviewer #1**: The objectives of this study are clear and well presented. The study design is appropriate and the population is clearly described, as well as the methods used. Appropriate statistical analyses were carried out. I have no ethical concerns with the study.

Line 101: The term ‘purified venom’ is a bit misleading since the term ‘purified’ usually refers to the use of isolated toxins. In this case the authors are using crude or whole venom. I suggest using the term ‘whole B. lanceolatus venom' throughout the paper instead of ‘purified venom’.

**Authors**: We agree that the term “purified venom” can be misleading. As suggested by the reviewer, we have used the term “whole *Bothrops lanceolatus* venom” throughout the revised manuscript.

Line 103: The authors only used one incubation time (24 hr). It would have been appropriate to use shorter incubation times as well, for example 3 hr or 6 hr, to have a view on the early effects of venom in the system. That would provide a time-course of the effects being studied.

**Authors**: We agree that providing time-course of the mitochondrial effects of whole B lanceolatus venom would be more appropriate than only measuring mitochondrial at a single point. Please understand that we had limited access to cardiac human biopsies that preclude full time-course evaluation. Regarding human cardiac tissue shortage, we have chosen to evaluate two different doses of venom instead of different incubation times. In our pilot experiments (N=2 at 3hr, N=2 at 6hr and N=2 at 12hr), we have determined that mitochondrial respiration deficits were apparent after 6hr on incubation). Such deficits were consistently observed at 24hr. Please find this information in the Results section of the revised manuscript.

**Reviewer #2**: Please refer general comments

**Results**

-Does the analysis presented match the analysis plan?
-Are the results clearly and completely presented?
-Are the figures (Tables, Images) of sufficient quality for clarity?

**Reviewer #1**: The analysis presented matched the analysis plan and are clearly formulated. Results are clear and well presented. The figures and tables are also clear and summarize the main findings of the study.

Line 174: The text is confusing with the use of the word ‘either’. Please revise.
**Authors**: We have revised the text according to reviewer’ comment.

**Reviewer #2**: Please refer general comments

**Conclusions**

-Are the conclusions supported by the data presented?
-Are the limitations of analysis clearly described?
-Do the authors discuss how these data can be helpful to advance our understanding of the topic under study?
-Is public health relevance addressed?

**Reviewer #1**: Most of the conclusions are supported by the data, with one exception: Line 226: The extrapolation of these observations to the clinical situation should be made with caution. In the experimental setting used. Cardiac tissue is directly exposed to venom for a prolonged period of time (24 hr). This is very different from a real envenoming case since (a) previous toxicokinetic data indicate that venom concentration in cardiac tissue after experimental envenoming in vivo is very low, and (b) the vasculature of the heart is of the ‘continuous’ type, meaning that it has low permeability and, therefore, the amount of venom that would reach myocardial cells is probably low in vivo. Thus, even though the observations clearly demonstrate that this venom is able to affect cardiac tissue and release DAMPs, the extrapolation of these findings to explain cardiac effects in snakebites is too speculative. This comment also applies to the conclusion paragraph (lines 248-250).
**Authors**: We agree that extrapolation of our results showing cardiac mitochondrial dysfunction is speculative. Accordingly, we have stated that “Further experiments using contracting isolated human right atrial trabeculae are warranted to demonstrate that such oxidative phosphorylation deficits may be associated with cardiac contractile dysfunction, which can occur in pitviper snake envenoming.” Likewise, we have modified the Conclusion section in the revised manuscript according to the comment of the reviewer.

The authors present a well-structured discussion on how these findings help to explain one important aspect of the action of snake venoms in tissues, i.e., the release of DAMPs which might impact on the innate immune response, with a possible effect on the systemic outcome of envenomings.
**Authors**: We thank the reviewer for its valuable comments.

The authors did not discuss the limitations of their work, some of which are: (1) the incubation time is 24 hr and it is not clear whether shorter time intervals would induce similar alterations. (2) The work was done with crude venom, and this does not allow the identification of the toxins responsible for the effects.
**Authors**: We have omitted to discuss main limitations of our study. Please find a specific section “Study limitations” in the revised manuscript.

Line 212: It should be Bothrops atrox, B. brazili and Lachesis muta

**Authors**: The manuscript has been revised according to reviewer’ comment.

Line 213: It should be ‘French overseas territories’

**Authors**: The manuscript has been revised according to reviewer’ comment.

**Reviewer #2**: Please refer general comments

**Editorial and Data Presentation Modifications?**

Use this section for editorial suggestions as well as relatively minor modifications of existing data that would enhance clarity. If the only modifications needed are minor and/or editorial, you may wish to recommend “Minor Revision” or “Accept”.

Reviewer #1: (No Response)
**Authors**: not applicable

Reviewer #2: Please refer general comments

**Summary and General Comments**

Use this section to provide overall comments, discuss strengths/weaknesses of the study, novelty, significance, general execution and scholarship. You may also include additional comments for the author, including concerns about dual publication, research ethics, or publication ethics. If requesting major revision, please articulate the new experiments that are needed.

**Reviewer #1**: This study presents an interesting contribution that expands our understanding on the action of the venom of B. lanceolatus. In recent years, the concept that snake venoms induce tissue damage with the release of DAMPs, and that these molecules may play a role in the overall pathophysiology of envenoming, contributing to systemic inflammation, has gained support. This study demonstrates with a sound experimental setting that the venom of B. lanceolatus is able to impair mitochondrial respiratory function in human cardiac tissue and also to release DAMPs from the tissue.
**Authors**: We thank the reviewer for its valuable comments.

My main concern has to do with the extrapolation made by the authors on the possible implications of their findings for the clinical cardiotoxicity induced by this venom. In my comments to the Discussion I have mentioned why I believe this extrapolation is too speculative and not necessarily supported by their findings. The main conclusion of this study should be focused on the ability of venom to impair mitochondrial function and to release DAMPS from the tissue, without extrapolating this to explain cardiac dysfunction in envenomings. For this, the authors should use in vivo experimental systems.
**Authors**: According to reviewer’ comments, extrapolation of our ex vivo findings to clinical cardiotoxicity induced by the whole B. lanceolatus venom has been omitted in the Discussion and Conclusion sections of the revised manuscript. The need to use in vivo experimental systems such as isometrically contracting isolated human right atrial trabeculae is mentioned in Discussion and Study Limitations sections of the revised manuscript

**Reviewer #2**: The study on Bothrops lanceolatus snake venom impairs mitochondrial respiration and induces DNA release in human heart preparation (PNTD-D-22-00263) by Cano-Sanchez et al have tried to address the effect of the venom on the cardiac muscle fibres, especially on the role of mitochondria in the venom induced toxicity. The authors have made an interesting observation of leaking of mitochondrial DNA which they consider it as the sole DAMP involved in venom induced cardiac related systemic toxicity. It is interesting that the authors have also tried to connect the mitochondrial DNA release to the innate immune response. The manuscript may be accepted after addressing the following queries.
**Authors**: We thank the reviewer for its valuable comments.

Minor comments:
Line 65 – remove receptor from “Toll-Like Receptor (TLR) receptors”.
**Authors**: The term receptor has been removed. Please read Toll-Like Receptors (TLRs)in the revised manuscript.

Line 103 - what is the vehicle control used?
**Authors**: We apologize for this omission. Cardiac cell preparations were incubated with commercial human serum albumin as controls. Pleased find this information in the Materials and Methods “Human atrial myocardium preparations” in the revised manuscript.

Line 135- mention the concentration of antimycin-A.
**Authors**: We apologize for this omission. The concentration of antimycin (2.5 μM) has been mentioned in the revised manuscript.

Line 192 - In table 2, third column doesn’t match the description of the legend.

**Authors**: We apologize for this typo error. The third column has been changed as required in the revised manuscript.

Line 195, 197,198 - There is no uniformity in manuscript writing, for example, VAA is written as VAA.

Line 196 - Correct the word ‘Vsucc+glu’.
We apologize for this typo error. “Vsucc+glu” has been changed to “Vsucc+glut” in the revised manuscript.

Line 106 – methodology for, ‘High-resolution respirometry’ is clumsy and difficult to follow. Split the methodology and explain in detail.
**Authors**: We have split and clarified the methodology for High resolution respirometry” in the revised manuscript.

Major comments:

What is the basis for Bothrops lanceolatus venom targeting cardiac muscle fibres only?
**Authors**: Our working hypothesis is that Bothrops lanceolatus venom would induce mitochondrial dysfunction, including membrane permeability increase leading to the release of mt DAMPs. We have previously used cardiac cell preparation in rodents and humans that display huge mitochondrial content (up to 30% of the cell volume). This specific characteristic provides a reasonable model to study the impact of Bothrops lanceolatus venom on mitochondrial function. Please find the statement “As mitochondria account for ∼35% of the cardiomyocyte volume, human heart preparations can provide a suitable model for the evaluation of the impact of snake venoms on mitochondrial function.” in the Introduction section of the revised manuscript.

The authors have to discuss about the fate of mitochondria after releasing their DNA. It is even better if they demonstrate the extent of their viability.
**Authors**: In cell physiology, reduction in mitochondrial DNA copy number is typically associated with the abnormal expression of oxidative phosphorylation (OXPHOS) system, resulting in mitochondrial dysfunction, then leading to perturbation on the electron transport chain and increased ROS generation and reduction in ATP level. Mitochondria fate will then be depending on the mitochondrial quality control regulation including mechanisms such as mitochondrial dynamics (fusion and fission), mitophagy and mitochondrial biogenesis. While these issues are of major interest in the area, the authors considered that these specific aspects beyond the scope in the present experiment work. Of note, we are currently testing in the laboratory whether snake venoms would induce mitochondrial perturbations such as ROS production, reduction in calcium retention capacity, and dissipation of the mitochondrial membrane potential.

Inclusion of the data on nuclear DNA release will make the readership better.
**Authors**: We agree with reviewer’ comment. As stated in the initial manuscript mtDNA levels was expressed relative to 18S gene used for the detection of nuclear DNA (Method and Figure 1).

As the study focuses on the mitochondrial dysfunction, it is essential to shed light on the possible role of mitochondrial membrane lipids, example lipid peroxidation, cardiolipin role etc if any.
**Authors**: We agree with reviewer’ comment. Whether and how snake venom would interact with membrane lipids and cardiolipin to alter mitochondrial function is clearly pertinent. Again, while these issues are of major interest in the area, the authors considered that these specific aspects beyond the scope in the present experiment work.

The authors have focused only on the release of mitochondrial DNA, it is important that they should address how and what promote the DNA release. Is the venom/venom toxin(s) exerting the DNA release effect directly interacting with the mitochondria (after crossing the sarcolemma) or does it acts at the sarcolemma level? An insight in to the mechanism of action will provide better clarity.
**Authors**: As we understand the model of permeabilized cardiac fiber model (our reference Kuznetsov et al. Analysis of mitochondrial function in situ in permeabilized muscle fibers, tissues and cells. Nat Protoc. 2008;3(6):965-76), the release of mtDNA may be directly from mitochondria. Owing to the presence of a hydrophobic steroid core, saponin has a high affinity to cholesterol and thus preferentially interact with cholesterol from membranes, specifically ‘attacking’ cholesterol-rich membranes, such as the sarcolemma leaving intracellular membrane structures (mitochondria and ER) as well as myofilaments and the cytoskeleton intact. Hence, it may be assumed that the venom/venom toxin(s) has directly alter membrane mitochondrial permeability.

It is important for the authors to demonstrate the state of respiration of mitochondria in venom treated cardiac fibers without saponin treatment.
**Authors**: Absence of saponin used will preclude adequate respiration analysis in response to substrates and/or inhibitors for respiratory experiments.

Mitochondrial DNA release and its connectivity to innate immunity need to be discussed properly.
**Authors**: We have included a specific paragraph related to “Mitochondrial DNA release and its connectivity to innate immunity in the Discussion section of the revised manuscript.

Please read in the revised manuscript: “Upon stress or cellular damage, one group of DAMPs, the mitochondrial nucleic acids, are released from their compartments and sensed as foreign, eliciting a similar innate immune response one would see against pathogens. The sensing of these molecules could be through intrinsic pathway or extrinsic pathway. In the intrinsic pathway, they are sensed in the cytoplasm of the cells undergoing the damage, while in the extrinsic pathway the sensing occurs outside in the plasma by other cells such as the dendritic cells or monocytes. Most of the sensors of mtDNA have only been recently identified. Amongst all the cytosolic DNA sensors, cyclic GMP–AMP synthase (cGAS), activating endoplasmic reticulum-resident stimulator of interferon genes (STING) are probably the most explored pathway. This pathway is triggered during infection with cytosolic bacterial pathogens and some DNA viruses resulting into transcriptional induction of type I interferon and the nuclear factor-κB (dependent expression of proinflammatory cytokines.”

PLOS authors have the option to publish the peer review history of their article ([what does this mean?](https://journals.plos.org/plosntds/s/editorial-and-peer-review-process#loc-peer-review-history)). If published, this will include your full peer review and any attached files.

If you choose “no”, your identity will remain anonymous but your review may still be made public.

**Do you want your identity to be public for this peer review?** For information about this choice, including consent withdrawal, please see our [Privacy Policy](https://www.plos.org/privacy-policy).

Reviewer #1: No

Reviewer #2: No

**Figure Files**

While revising your submission, please upload your figure files to the Preflight Analysis and Conversion Engine (PACE) digital diagnostic tool, https://pacev2.apexcovantage.com. PACE helps ensure that figures meet PLOS requirements. To use PACE, you must first register as a user. Then, login and navigate to the UPLOAD tab, where you will find detailed instructions on how to use the tool. If you encounter any issues or have any questions when using PACE, please email us at figures@plos.org.
 
Data Requirements:

Please note that, as a condition of publication, PLOS' data policy requires that you make available all data used to draw the conclusions outlined in your manuscript. Data must be deposited in an appropriate repository, included within the body of the manuscript, or uploaded as supporting information. This includes all numerical values that were used to generate graphs, histograms etc.. For an example see here: http://www.plosbiology.org/article/info%3Adoi%2F10.1371%2Fjournal.pbio.1001908#s5.
 
Reproducibility:

To enhance the reproducibility of your results, we recommend that you deposit your laboratory protocols in protocols.io, where a protocol can be assigned its own identifier (DOI) such that it can be cited independently in the future. Additionally, PLOS ONE offers an option to publish peer-reviewed clinical study protocols. Read more information on sharing protocols at https://plos.org/protocols?utm_medium=editorial-email&utm_source=authorletters&utm_campaign=protocols

__________________________________________________
In compliance with data protection regulations, you may request that we remove your personal registration details at any time. (Use the following URL: https://www.editorialmanager.com/pntd/login.asp?a=r). Please contact the publication office if you have any questions.
